# Model predicts coastal paralytic shellfish toxicity in mussels from in situ phytoplankton concentrations

**DOI:** 10.1002/lno.70502

**Published:** 2026-09-14

**Authors:** Sylvain Gaillard, David K. Ralston, V. Monica Bricelj, Roman Marin, Bruce Keafer, Dennis J. McGillicuddy, Donald M. Anderson

**Affiliations:** ^1^ Woods Hole Oceanographic Institution Woods Hole Massachusetts USA; ^2^ TechMar Research Inc. Brooklyn New York USA; ^3^ Monterey Bay Aquarium Research Institute Moss Landing California USA

## Abstract

Harmful algal blooms (HABs) of *Alexandrium catenella* occur annually in the Gulf of Maine, leading to accumulation of paralytic shellfish toxins (PSTs) in bivalve mollusks and closure of harvesting areas. State monitoring programs in the region rely on weekly shellfish sampling and toxin analysis, requiring extensive effort with limited temporal resolution. With the development of autonomous in situ detection devices such as the Environmental Sample Processor (ESP), this study aimed to model shellfish toxicity using ESP‐derived *A*. *catenella* counts to complement traditional monitoring programs using mussels (*Mytilus edulis*). We developed a one‐compartment model of PST accumulation using *A. catenella* concentrations from nearshore ESP deployments. Multi‐year and multi‐site data were used to test four approaches for estimating mussel toxin uptake and depuration parameters, that is, local, global, or fixed literature values. When compared with direct PST measurements in nearby shellfish, the models achieved high correlations (*R* ^2^ = 0.69–0.90), with no false negatives. Model fit varied mainly with toxin uptake parameters and was influenced by *Alexandrium* concentrations. The impact of other environmental and biological factors on uptake and depuration parameters remained elusive, likely due to the mussels' physiological plasticity, spatial or hydrographic separation between ESP and toxicity measurements, and microhabitat conditions. This ESP‐based model offers a simple, effective tool for translating offshore, automated cell counts into daily shellfish toxicity estimates and is promising for improvement of monitoring capacity in remote or under‐sampled areas. Incorporation of this approach into physical–biological models and application in early warning systems with other biosensors remain to be tested.

Harmful algal blooms (HABs) are an important problem affecting marine and freshwater coastal waters worldwide (Smayda 1997). The most significant and widespread impacts of HABs are the poisoning syndromes that arise when humans consume shellfish, especially suspension‐feeding bivalves, which accumulate HAB toxins in their tissues (Bricelj and Shumway 1998). Toxic species of the genus *Alexandrium* form HABs in coastal and estuarine waters, and they can produce paralytic shellfish toxins (PSTs, also referred to as saxitoxins) responsible for paralytic shellfish poisoning (PSP) in humans (Anderson et al. 1990). Individual PSTs are classified according to their toxicity to mice, and the most potent are the so‐called carbamoyl toxins, namely saxitoxin (STX), neosaxitoxin (neoSTX), and gonyautoxins (GTXs) (EFSA 2009).

The cyst‐forming dinoflagellate *Alexandrium catenella* (Whedon & Kofoid) Balech 1985, is the most widespread PST‐producing species on both Atlantic and Pacific coasts of the US (Anderson et al. 2012a). The Gulf of Maine (GoM) experiences nearly annual blooms of *A. catenella* that have been associated with shellfish toxicity for over 40 yr (Shumway et al. 1988; Anderson et al. 2021a). Blooms of *A. catenella* in the GoM originate from cyst germination at two major cyst accumulation zones, one located in the Bay of Fundy and another off the mid‐coast of the state of Maine (Anderson et al. 2005). Germinated motile cells are transported along the coast from the northeast to the southwest by the Maine Coastal Current (Anderson et al. 2005).

As a result of public health concerns, US state and federal agencies have instituted a range of monitoring and management policies to keep toxic shellfish off the market (Shumway et al. 1988). Large expanses of coastline are often closed seasonally (e.g., annual closures from May to October for PSP in Maine; Anderson et al. 2014), or for longer periods for recreational or subsistence harvesting of shellfish in Alaska due to PSP (Anderson et al. 2021b). Monitoring involves the collection of shellfish from coastal stations, typically on a weekly basis, and testing of soft tissue extracts for toxicity to guide regulatory decisions. Thus, the state of Maine has monitored as many as 160 stations annually for PSP toxicity (Shumway et al. 1988; Grasso et al. 2019). Monitoring programs typically use mussels (e.g., the blue mussel *Mytilus edulis*) as sentinel organisms for PSP due to their widespread distribution, capacity to rapidly accumulate high toxicities, and relatively fast depuration, compared to other bivalves (Bricelj and Shumway 1998).

The policies and procedures for phytoplankton monitoring and shellfish flesh testing detailed in national and international guidelines (National Shellfish Sanitation Program 2017; EU Commission 2011) have changed little over the years. However, these techniques are labor‐ and time‐intensive, requiring transportation of samples from the field to the laboratory for subsequent analysis, which can take days to be completed, thus reducing the frequency and extent of the sampling (Anderson et al. 2012b).

In addition to toxin monitoring, numerical modeling is used to provide bloom outlooks that are useful in managing public health risks from HABs. For example, a physical–biological model coupling hydrodynamics and *A. catenella* cyst distribution and bloom dynamics in the GoM has been used to forecast cell concentrations of the dinoflagellate (McGillicuddy et al. 2011; Flynn and McGillicuddy 2018). However, Record et al. (2022) highlighted specific needs for the prediction of toxicity in shellfish rather than *A. catenella* concentration, particularly for local stakeholders such as shellfish farmers and resource managers. To improve the accuracy of such models for shellfish toxicity and provide early warning of toxicity events, real‐time sampling of HAB species cell abundance and toxicity is required (Anderson et al. 2013).

Recent decades have seen great advances in the technologies used to rapidly detect and quantify HAB cells and their toxins, helping to expand traditional monitoring strategies (Sellner et al. 2003; Anderson et al. 2012b, 2015). Novel approaches to measure these parameters have been incorporated into robotic, autonomous biosensors that can be moored in coastal waters, providing nearly real‐time data from remote locations (Davidson et al. 2016; Moore et al. 2021). For example, the Environmental Sample Processor (ESP) is a robotic electromechanical/fluidic system that can automatically collect and filter water samples and use DNA and antibody‐based probes to detect and quantify certain HAB cells and their toxins (Doucette et al. 2009; Scholin et al. 2017; Moore et al. 2021). ESPs have been integrated into an autonomous underwater vehicle to quantify *Microcystis* sp. in Western Lake Erie in the US (Den Uyl et al. 2022) and deployed on moorings on the Pacific Northwest coast of the US to monitor blooms of HAB species in situ, including cells of *Pseudo*‐*nitzschia* spp. and *A. catenella*, as well as domoic acid (Moore et al. 2021).

The PST content in suspension‐feeding bivalves depends on the balance of several physiological processes, including uptake of toxic cells (a function of clearance rates by the gills, and selective ingestion), absorption rate of algal cells in the digestive gland, toxin tissue compartmentalization, biotransformation, and depuration, influenced by environmental conditions such as temperature, cell density, and algal specific toxicity (Blanco 2009). Various accumulation models for microalgal toxins in mollusks have been developed to represent the mechanisms of uptake and depuration, with some incorporating additional parameters for toxin compartmentalization (Silvert and Cembella 1995) and biotransformation (Blanco et al. 2003) in tissues as well as dependence on environmental variables (Blanco et al. 1997). Most of the models describe PST accumulation for mussels (*Mytilus* spp.) exposed to blooms or cultures of *Alexandrium* spp. or *Gymnodinium catenatum* (Supporting Information Table S1). These studies generally integrate microscopically‐determined cell counts as well as one value of toxicity (sum of all the toxin congeners in the case of PSTs, with toxicity expressed in *μ*g STX equivalents (eq.)) and use a one‐compartment model (i.e., whole tissues), with model parameters (uptake and depuration rates) obtained either from laboratory or field‐based measurements. The two‐compartment model assumes that toxins originating from the ingested phytoplankton are accumulated in a first compartment (digestive organs) where they are labile, that is, weakly bound, and are transferred to a second compartment (other tissues), where toxins are more strongly bound (Silvert and Cembella 1995). Model parameters are fitted to observed data (Supporting Information Table S1).

The present study describes a novel approach in which *A. catenella* concentrations are measured with ESP technology at mooring locations in the GoM and used to model shellfish toxicities measured in mussels at nearby coastal monitoring stations. Multiple ESP data series over multiple years are compared with Maine monitoring program PSP shellfish toxicity measurements to develop a toxicity model. We also assess the potential role of ESP‐based models to complement traditional PSP shellfish monitoring programs by providing higher‐frequency, higher‐resolution data products to inform decision making. Finally, we discuss incorporation of the toxicity model into a physical–biological model of HAB cell abundance, as suggested by Anderson et al. (2013).

## Materials and methods

### Study area

**Fig. 1:**
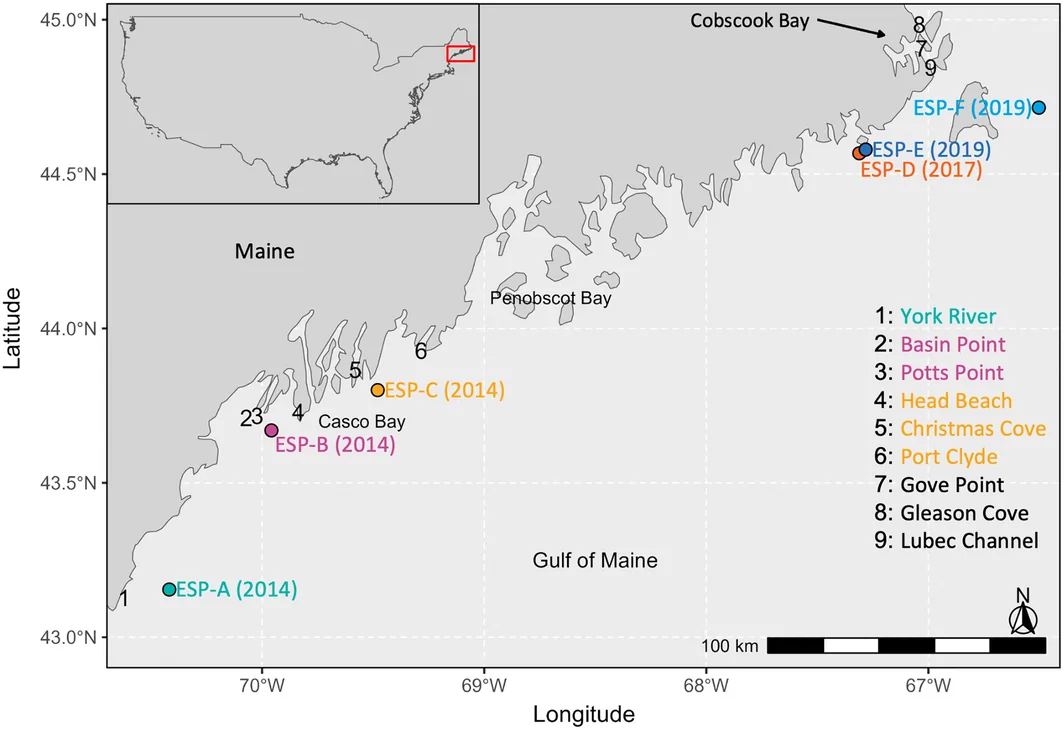
Map of the Gulf of Maine showing deployment locations of the Environmental Sample Processors (ESPs) and paired Maine Department of Marine Resources (DMR) mussel (*Mytilus edulis*) sampling areas (*n* = 15 ESP‐toxicity sampling station pairs) from 2014 to 2019. Paired ESPs and stations are represented in the same color. ESP‐D, E, and F are each paired with stations 7, 8, and 9.

ESPs were deployed from late spring to late summer from 2014 to 2019 at several locations in coastal Maine waters (Fig. 1; Supporting Information Table S2). In 2014, ESPs were deployed at three locations along the Western Maine coast, offshore of York River (ESP‐A) and near Casco Bay (ESP‐B and ESP‐C). In 2017 and 2019, ESPs were deployed near the mouth of the Bay of Fundy, in coastal waters offshore of Cobscook Bay and Grand Manan Island (ESP‐D in 2017; ESP‐E and ESP‐F in 2019). The locations were selected to be as near to Maine shellfish monitoring stations as possible given depth constraints associated with the mooring system. Observational monitoring efforts were focused on Casco Bay and Cobscook Bays due to their frequent toxicity events (Shumway et al. 1988; Thomas et al. 2010).

### Blue mussel (*Mytilus edulis*) sampling and toxicity analysis

Mussels were collected weekly by the Maine Department of Marine Resources (DMR) during routine monitoring at stations along the coastline (Fig. 1; Supporting Information Table S3) from the intertidal zone or from cages deployed at locations with low mussel abundances. Pooled tissues from a minimum of 12 adult mussels that met a minimum size of 2″ (5 cm) representative of marketable mussels (https://www.maine.gov.dmr/files/docs/12.pdf) were analyzed for PST concentrations by Bigelow Analytical Services (Bigelow, East Boothbay, Maine, USA) using high‐performance liquid chromatography with fluorescence detection (HPLC‐FLD), as described by Grasso et al. (2019).

### Environmental Sample Processor deployments

The ESP is a submersible, robotic instrument that collects discrete water samples and applies molecular probes to identify target microorganisms and gene products (Scholin et al. 2017). Samples are processed in robotically manipulated reaction chambers (pucks), using ribosomal RNA‐targeted DNA probes that are specific to *A. catenella* (Greenfield et al. 2008) in sandwich hybridization assays (SHA; Greenfield et al. 2006). The concentration of target nucleic acids in the collected sample is determined by comparison to a laboratory‐maintained *A. catenella* culture at cell densities of 0, 78, 156, 312.5, 625, 1250, and 2500 cells mL^−1^. ESP detection of *A. catenella* has been demonstrated in multiple field studies (Greenfield et al. 2008; Moore et al. 2021).

ESPs were deployed on moorings with a surface buoy that provided real‐time communications to shore. They were connected to the surface buoy with a high‐stretch mooring cable and submerged at 20 m depth to account for the large tidal range in the GoM and to insulate the instruments from high‐energy surface waves. Near‐surface seawater samples were collected at the base of the surface buoy (~ 1 m depth) and pumped down to the ESP for analysis. Near‐surface sampling may be influenced by vertical migration of *A. catenella* cells. However, the capacity to perform vertical migration is not universal among GoM strains, and the importance of this behavior has not yet been demonstrated for regional populations (Townsend et al. 2005). Four (in 2014) or two liters (2017 and 2019) of seawater were collected using the onboard ESP 25 mL syringe pump over 75 (in 2014) or 40 min (in 2017 and 2019). Sample water was filtered through a 10 *μ*m titanium frit, and particulates collected on the frit were then lysed with ESP lysis buffer provided by the Monterey Bay Aquarium Research Institute. Due to limitations on the total number of samples during each deployment, the ESPs sampled once per day for five consecutive days, skipped 2 d, then resumed daily sampling for the next 5 d, and so on. Because tidal currents were strong, all samples were collected at slack tide to limit mooring‐line and instrument tilt, which if excessive could hinder instrument operation. Sampling alternated between slack before flood and before ebb tide to minimize biases.

### PST accumulation model

The model used in this study is a one‐toxin group (PSTs) and one‐compartment model (Silvert and Cembella 1995; Blanco 2009), assuming no transformation between individual toxins, formulated as follows:

(1) $$\frac{dT}{dt}=\mathrm{\beta C}\left(t\right)-\mathrm{\gamma T}\left(t\right)$$

where T is the toxicity of *M. edulis* at the Maine DMR monitoring stations (*μ*g STX eq. 100 g^−1^ soft tissue), t is time in days, C is the *A. catenella* concentration estimate from the ESP (cells L^−1^), β is the toxin uptake parameter [*μ*g STX eq. 100 g^−1^ soft tissue (L cell^−1^) (d^−1^)] and γ is the depuration or toxin elimination rate (d^−1^) of *M. edulis*. The uptake was standardized by the cell concentration. The daily toxin uptake rate of *M. edulis* is the product of β and the local *A. catenella* cell concentration.

In Eq. 1, *A. catenella* cell concentrations measured by the ESPs are model inputs but the uptake (β) parameter and depuration (γ) rate are unknown and determined based on fitting to the observed mussel toxicity data. The ESPs were paired with nearby toxicity monitoring stations to test the model (Supporting Information Table S4). The optimal β and γ values are determined by testing a range of values for each ESP‐toxicity paired station and selecting the parameter combination that provides minimum error between modeled and observed toxicity. An optimal parameter combination can be determined for each ESP‐toxicity station pair independently, and this is called the local fit. Alternatively, we can find the β and γ parameters combination that provides the minimum error combining all 15 of the ESP‐toxicity stations together, and this provides the global fit. The local fits have more free parameters and thus result in lower errors than the individual pairs, but the global fit may be more broadly applicable under the assumption that the dynamics linking offshore cell concentrations with shellfish toxicity at monitoring stations are common among the different locations.

The depuration or toxin elimination rate (γ) depends primarily on the bivalve's metabolic processes and is relatively constant for a given species, although it was found to be temperature‐dependent in surf clams *Spisula solidissima* (Bricelj et al. 2014). Based on published laboratory and field studies of one or two‐compartment toxicity models, we found that the reported depuration rate of PSTs in *Mytilus* averages (± standard deviation, SD) 0.19 ± 0.11 d^−1^ (Supporting Information Table S1). In addition, several studies used a constant depuration rate of 0.15 d^−1^ and highlighted optimal results using this value (Blanco et al. 2003, 1997; Moroño and Blanco 1997; Supporting Information Table S1). Many studies report the mussels' clearance rate (volume of water cleared of particles per unit of time) rather than directly measuring STX uptake, but we estimate the range of published uptake parameters as 4 × 10^−6^ to 3 × 10^−2^ *μ*g STX eq. 100 g^−1^ tissue L cell^−1^ d^−1^ (Supporting Information Table S1). We tested the model using a fixed depuration rate of 0.15 d^−1^ and fit to the observations by adjusting only the uptake parameters, which are less well constrained in the literature than depuration rates. Using this fixed depuration rate of 0.15 d^−1^, the uptake parameters are fit locally to each ESP‐toxicity station pair as well as globally by minimizing the error for all pairs together.

The model cases are numbered for reference as follows: Model 1 (M1) uses local fits of β and γ (most free parameters); Model 2 (M2) uses a global fit of β and γ; Model 3 (M3) uses local fits for β with a fixed γ = 0.15 d^−1^; and Model 4 (M4) finds a global fit of β with a fixed γ = 0.15 d^−1^ (fewest free parameters) (Table 1). Parameter fitting was done by running the model for each ESP‐toxicity station pair using a range of values for β (0–0.8 *μ*g STX eq. 100 g^−1^ tissue L cell^−1^ d^−1^, increment of 0.03) and γ (0.01–0.4 d^−1^, increment of 0.02). The models were initialized when mussel toxicities at the DMR sampling locations are first reported above detection to focus on the bloom period. Models were run forward in time using the observed ESP cell concentrations as input. The optimal parameter values were found based on minimizing the mean square error (MSE) between the modeled and observed toxicity. For the local models (M1 and M3), parameter values were found for each ESP‐toxicity station pair. For the global models (M2 and M4), parameter values were determined by minimizing the combined MSE for all station pairs. Pearson's correlation coefficient squared or coefficient of determination (*R* ^2^) and statistical significance (*p*‐value) of the correlation were also calculated for each of the fits. To account for the differences in free parameters among the models, the Akaike Information Criteria (AIC) was calculated to evaluate the tradeoff between goodness of fit and model complexity. With the four described approaches to fitting the toxicity model we can evaluate the tradeoffs in degrees of freedom vs. goodness, simplicity and/or applicability of fit.

**Table 1:** Model parameters and metrics used in this study. *R* ^2^ (Pearson's correlation coefficient squared) and Akaike Information Criteria (AIC). For the local models (M1 and M3), parameter values are the means of individual fits for ESP‐toxicity station pairs, with the range in parentheses. For M3 and M4, the depuration rate is fixed as γ = 0.15 d^−1^. M0: Model 0; M0L: Model 0 lagged; M1: Model 1; M2: Model 2; M3: Model 3; M4: Model 4; ***p ≤ 0.001. n.a.: not available.

Model	Uptake (β) *μ*g STX eq. 100 g^−1^ tissue L cell^−1^ d^−1^	Depuration (γ) d^−1^	*R* ^2^	AIC
M0	n.a.	n.a.	0.12***	n.a.
M0L	n.a.	n.a.	0.45***	n.a.
M1	0.032 (0.009–0.066)	0.16 (0.03–0.35)	0.90***	286
M2	0.021	0.11	0.71***	278
M3	0.030 (0.012–0.072)	0.15	0.85***	167
M4	0.027	0.15	0.69***	204

For comparison, we also assessed the correlation between measured *A. catenella* concentrations (cells L^−1^) and mussel toxicities (*μ*g STX eq. 100 g^−1^ tissue). This is in effect a simpler model for toxicity, neglecting uptake and depuration dynamics and instead assuming that the mussels' toxin body burden is linearly proportional to *A. catenella* abundance. This is justified because *M. edulis* is relatively insensitive and maintains high ingestion on *A. catenella* up to ~ 10^5^ cells L^−1^ (Bricelj et al. 1990) (> than an order of magnitude above the maximum environmental observed concentrations in the present study). We evaluate this direct correlation with the cell concentration (Model 0, M0) and as a lagged correlation based on the maximum *R* ^2^ over lags of 1–7 d (Model 0 lagged, M0L).

## Results

### *Alexandrium catenella* and mussel toxicity field measurements

Of the 36 ESP‐toxicity sampling station pairs analyzed in this study, 15 were considered suitable for model analysis. These pairs included: (1) stations where measured toxicities were ≥ 40 *μ*g STX eq. 100 g^−1^ soft tissue (i.e., a moderate toxin level of half of the regulatory limit (RL) for STX in shellfish (Lefebvre et al. 2022)) and (2) stations located along the open coast rather than in inshore embayments, which are less connected to the coastal ocean where the ESPs were moored. The exclusion of embayment stations also aimed to minimize confounding effects from localized blooms and/or cyst resuspension events, which are common in these semi‐enclosed habitats.

**Fig. 2:**
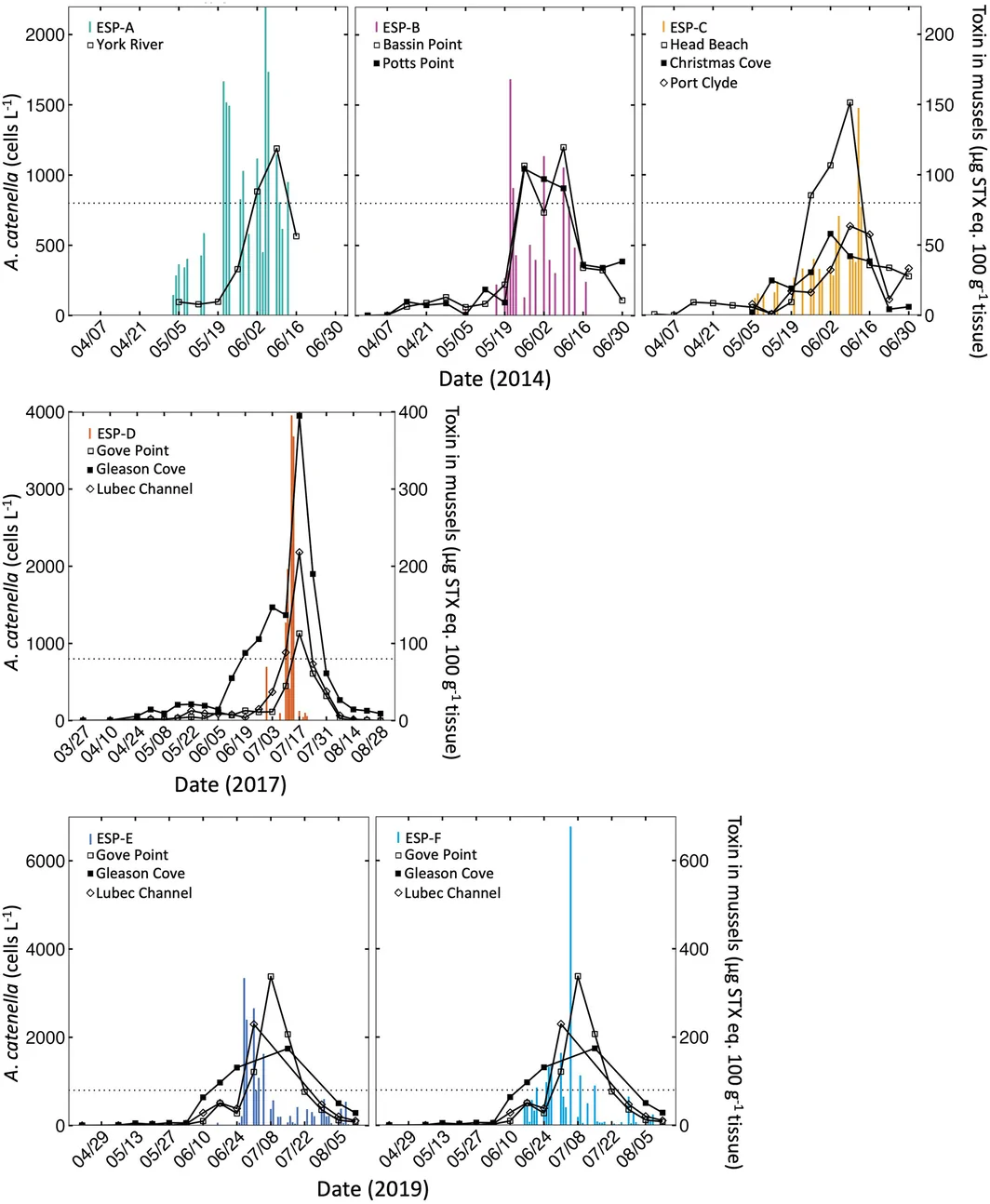
Time series (2014–2019) of Environmental Sample Processor (ESP) measurements of *Alexandrium catenella* concentrations (bars; cells L^−1^) and paralytic shellfish toxin (PST) toxicities in *M. edulis* tissues [lines; *μ*g saxitoxin (STX) equivalents (eq.) 100 g^−1^ soft tissue] sampled at the paired Maine Department of Marine Resources (DMR) stations in coastal Maine. Dashed line represents the regulatory limit (80 *μ*g STX eq. 100 g^−1^ soft tissue).

For the 15 pairs analyzed (Fig. 2), *A. catenella* concentrations measured by the ESP had 90^th^ percentile values ranging from 556 to 1505 cells L^−1^, with a maximum value of 6750 cells L^−1^ recorded by ESP‐E in 2019 (Fig. 2). Initial ESP detection of *A. catenella* in 2014 occurred in early May, whereas blooms were detected later in 2017 (late June) and 2019 (early July). Elevated cell concentrations generally persisted for 1–2 months (Fig. 2). At the toxicity monitoring stations paired with these ESPs, 14–17% of the PST measurements in mussels were above the RL (i.e., 80 *μ*g STX eq. 100 g^−1^ soft tissue), depending on the year (Fig. 2).

### Modeled toxicity in mussels

The simplest models, that is, M0 and M0L, showed a moderately high correlation (i.e., *R* ^2^ > 0.70) between *A. catenella* cell concentrations and PST toxicity for a subset of ESP‐toxicity station pairs: 4 out of 15 pairs for M0 and 9 out of 15 pairs for M0L (Table 1; Supporting Information Table S4). However, the overall correlations of mussel toxicity observations with *A. catenella* cell concentrations, with or without a time lag, were relatively low (*R* ^2^ = 0.12 and 0.45 for M0 and M0L, respectively; Table 1; Supporting Information Table S4; Supporting Information Fig. S1).

**Fig. 3:**
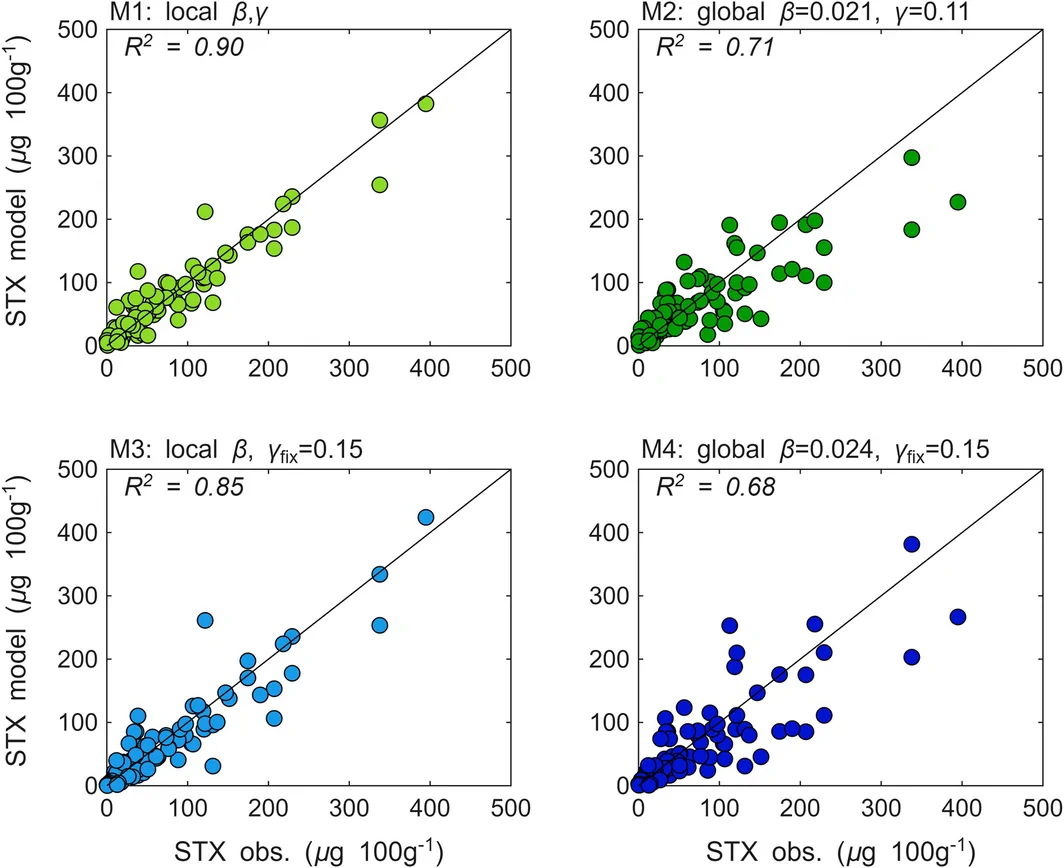
Correlation plots of the observed and modeled mussel toxicities [*μ*g saxitoxin (STX) equivalents (eq.) 100 g^−1^ tissue] for the four different models (Table 1). One‐to‐one lines are shown in black for reference. β: toxin uptake parameter; γ: toxin depuration parameter. M1, M2, M3, and M4: Models 1 through 4.

The one‐compartment toxicity models incorporating uptake and depuration yielded higher correlations between observed and modeled PST concentrations than M0 and M0L, with an overall *R* ^2^ of 0.69–0.90 for the different cases (Fig. 3; Table 1; Supporting Information Table S4). Models with local fits for each ESP‐toxicity station pair had the strongest correlations with an overall *R* ^2^ of 0.90 for M1 and 0.85 for M3, and *R* ^2^ values of individual fits ranging from 0.33 to 0.99. M3 was the best‐supported model based on having the lowest AIC value (Table 1). The global fits that grouped all the ESP‐toxicity station pairs together had an overall *R* ^2^ of 0.71 for M2 and 0.69 for M4, with individual fits ranging from 0.16 to 0.98 (Fig. 3; Table 1; Supporting Information Table S4). No significant relationship was observed between model fits and distance between ESP and mussel sampling locations (Supporting Information Table S4).

**Fig. 4:**
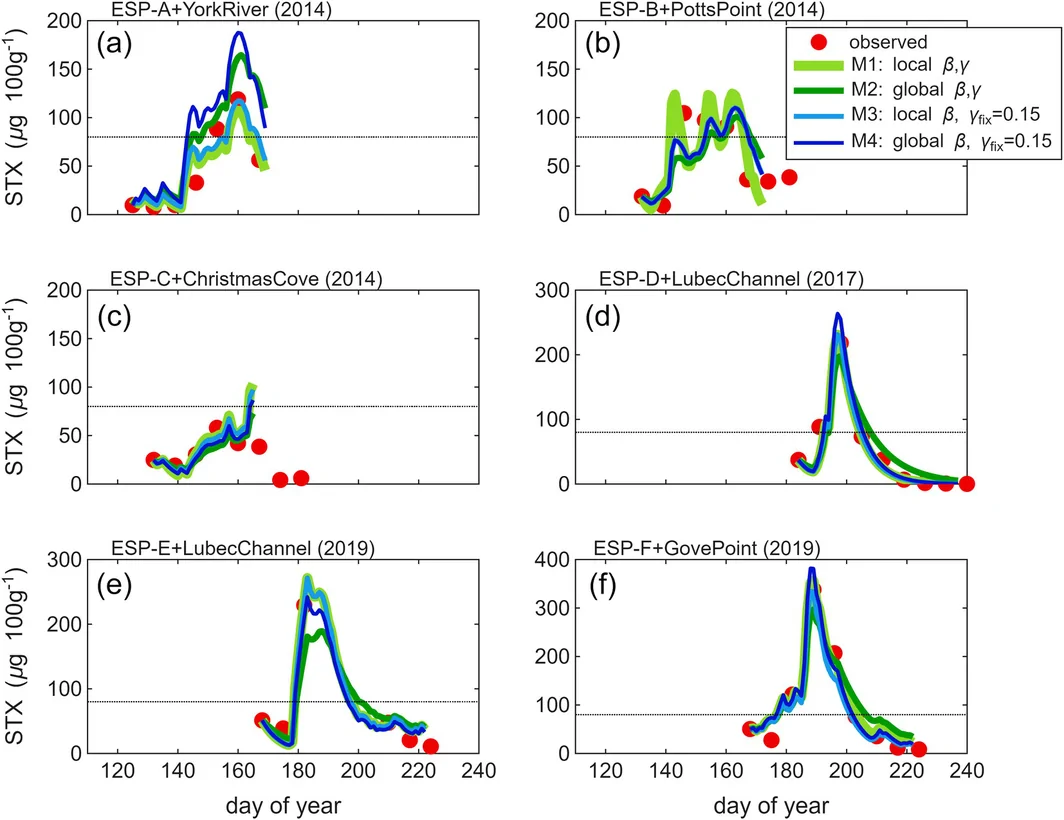
Selected time series of observed paralytic shellfish toxins (PST) in blue mussel (*Mytilus edulis*) tissues [*μ*g saxitoxin (STX) equivalents (eq.) 100 g^−1^ soft tissue] sampled at coastal Maine Department of Marine Resources (DMR) stations (Fig. 1), and modeled toxicity (*μ*g STX eq. 100 g^−1^ soft tissue) (Table 1; Supporting Information Table S4). Other examples of good fits are presented in Supporting Information Fig. S2. Horizontal dashed line represents the regulatory limit (80 *μ*g STX eq. 100 g^−1^ soft tissue). β: toxin uptake parameter; γ: toxin depuration parameter; ESP: Environmental Sample Processor; M1, M2, M3, and M4: Models 1 through 4.

When individual ESP–toxicity station pairs were compared (Table 1; Supporting Information Table S4), the models successfully used nearly daily *A. catenella* concentrations to estimate daily mussel PST concentrations between the weekly toxicity measurements (Fig. 4; Supporting Information Fig. S2). The results suggest that toxicity could have exceeded the RL for multiple days in between the weekly mussel sampling in 2014 at Potts Point (Fig. 4b) and Basin Point (Supporting Information Fig. S2a) and in 2019 at Gove Point (Fig. 4f). The global models (M2 and M4) overpredicted toxicities for several days in 2014 for ESP‐A at York River, indicating toxicity levels above the RL and potentially false positives (Fig. 4a). Importantly, no false negatives were detected in the 15 pairs. In some cases, toxicities below the RL were still detected in mussels even when *A. catenella* was not detected by the ESP (e.g., 2014 ESP‐D Gleason Cove; Fig. 2; Supporting Information Fig. S1).

### Uptake and depuration parameters

Toxin uptake parameters (β) resulting from the local fits had similar mean values (± SD) of 0.032 ± 0.020 for M1 with locally fitted depuration and 0.030 ± 0.015 *μ*g STX eq. 100 g^−1^ tissue L cell^−1^ d^−1^ for M3 with fixed depuration, with uptake parameters for individual fits ranging by one order of magnitude (Table 1). The global fits yielded 1.2 to 1.5‐fold lower uptake parameters with β = 0.021 for M2 and β = 0.027 *μ*g STX eq. 100 g^−1^ tissue L cell^−1^ d^−1^ for M4 with fixed depuration (Table 1).

**Fig. 5:**
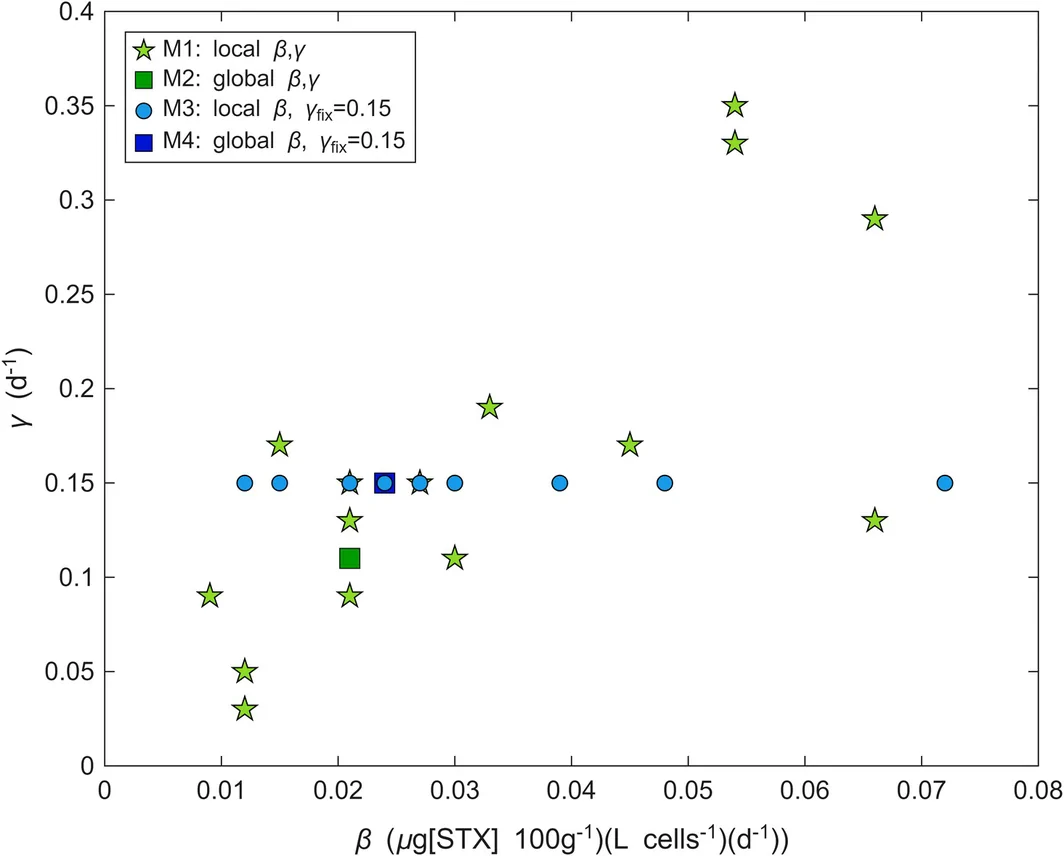
Toxin uptake (β) and depuration (γ) parameters obtained for the four different models (Table 1). For Model 3, there are only nine unique values of toxin uptake. STX: saxitoxin; M1, M2, M3, and M4: Models 1 through 4.

Toxin depuration parameters (γ) from the local fits (M1) averaged 0.162 ± 0.095 d^−1^ and ranged from 0.03 to 0.35 (Fig. 5; Table 1; Supporting Information Table S4). The depuration parameter for the global fit M2 was 0.11 d^−1^. These depuration values derived from the model fitting were similar to the fixed depuration value of 0.15 d^−1^ used in M3 and M4 and are consistent with published values reported for mussels (e.g., Blanco et al. 2003, 1997; Moroño and Blanco 1997; Supporting Information Table S1). Among the cases where both β and γ were free parameters (M1 and M2), there was a general trend of covariance, with greater uptake parameters associated with greater depuration rates (Fig. 5). This covariance in β and γ parameters is found among the ESP‐toxicity station pairs, and also within the parameter pairs tested in the optimization, as is examined next.

**Fig. 6:**
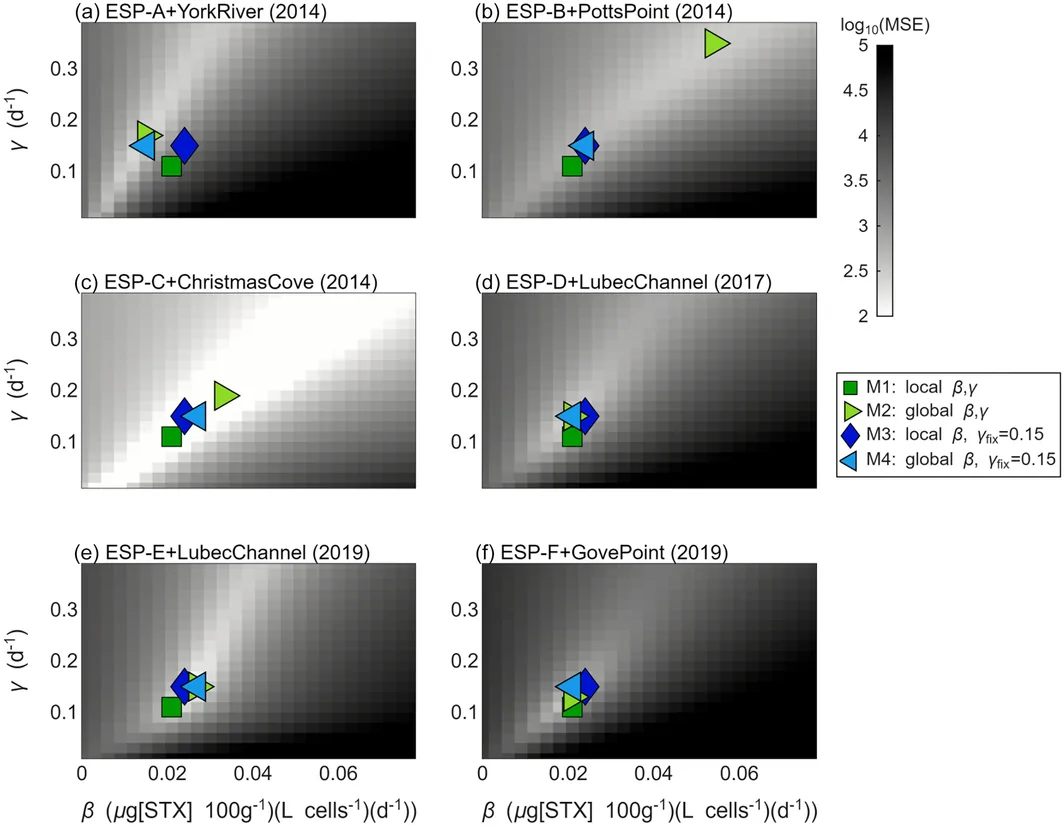
Selected correlation plots of toxin uptake (β; *μ*g saxitoxin (STX) equivalents (eq.) 100 g^−1^ tissue L cell^−1^ d^−1^) and depuration (γ; d^−1^) parameters, and log_10_ mean squared error (MSE). ESP: Environmental Sample Processor; M1, M2, M3, and M4: Models 1 through 4.

In fitting the models where both β and γ parameters are assumed to be unknown (M1 and M2), we find that there are regions of parameter space where multiple, co‐varying values of the two parameters have a similarly low mean squared error (MSE; Fig. 6). For the local fits, we can select a parameter set that provides the minimum MSE, but these values are not well constrained since proportional increases or decreases in β and γ can provide similarly low errors. Typically, the global fit β and γ (M2) as well as the fixed depuration rate models (M3 and M4) fell in or near the region of parameter space with low MSE for the local fits of M1.

## Discussion

This study successfully linked *A. catenella* concentrations determined in Maine coastal waters using autonomous robotic samplers, that is, ESPs, with weekly blue mussel toxicity data from Maine DMR monitoring stations using a one‐compartment model. Correlations (R^2^) were high overall, decreasing from 0.90 to 0.69 with the increasing number of free parameters (Table 1; Supporting Information Table S4).

Blue mussels provide the simplest case for our modeling approach compared to other suspension‐feeding bivalves, thereby reducing variability in toxin uptake. Indeed, they exhibit low ingestion selectivity when exposed to toxic phytoplankton, in contrast to Eastern oysters (*Crassostrea virginica*), which show clearance rate inhibition and strong particle selection (Beninger et al. 2004; Mafra et al. 2009a, 2009b), and more limited biotransformation of PSTs than bivalves such as *S. solidissima*.

Analysis of the relationship between daily *A. catenella* concentrations determined using the ESPs and mussel toxicities showed a variable linear correlation among locations (overall *R* ^2^ = 0.12, range = 0.01–0.97; M0; Table 1; Supporting Information Table S4). Similarly, in Puget Sound elevated PST shellfish toxicities were typically preceded by an increase in *A. catenella* concentrations (measured by qPCR), although high *A. catenella* cell densities occasionally occurred without subsequent toxicity outbreaks (Dyhrman et al. 2010). Such inconsistencies between cell concentrations and toxicity in shellfish suggest a lag time, ranging from days to weeks (Bricelj and Shumway 1998). While the inclusion of a temporal lag in this study resulted in slightly better fits, the overall *R* ^2^ remained relatively low (*R* ^2^ of 0.45; M0L; Table 1; Supporting Information Table S4). Future efforts could refine lag‐time estimates relevant to the ESP‐toxicity approach through the use of circulation models and autonomous sampling devices deployed offshore and near mussel leases.

The limited correspondence between cell concentrations and mussel toxicity data motivated the development of a toxicity model to better capture toxin uptake and depuration processes and accurately resolve the HAB‐shellfish toxicity relationship. Model optimization approaches that calculated local parameters for each ESP‐toxicity station pair (M1 and M3) exhibited high correlations (overall mean *R* ^2^ ≥ 0.85; Table 1; Supporting Information Table S4). Models with the global fits using all the ESP‐toxicity station pairs together (M2 and M4) yielded lower, moderate correlations (overall mean *R* ^2^ > 0.69; Table 1; Supporting Information Table S4) but may be more suitable for broader application.

Prior applications of one‐compartment models (Table S1) found correlations similar to those in this study, with *R* ^2^ values from 0.45 (Moroño and Blanco 1997) to > 0.80 (Braga et al. 2018; Tang et al. 2021). These models have been employed for their simplicity and reasonable fits (Blanco et al. 2003), and they provide a “useful representation” of toxin dynamics in the natural environment (Silvert and Cembella 1995).

### Factors influencing model fit

The use of two‐compartment models has been shown to improve the fit of mussel toxicities compared to one‐compartment models (e.g., a twofold increase in *R* ^2^; Moroño and Blanco 1997; Table S1). The two‐compartment model is particularly useful when biphasic depuration is observed, that is, when the modeled toxicity decreases faster than the observed toxicity (Silvert and Cembella 1995; Blanco 2009). However, Bricelj and Shumway (1998) showed that the bulk of PSTs in contaminated *M. edulis* are localized in the digestive gland during both exposure and depuration phases, thus supporting the use of a one‐compartment model for this species, in contrast to other bivalves such as the butter clam *Saxidomus giganteus* and surf clam *S. solidissima*. In some instances, background toxicities (< 40 *μ*g STX eq. 100 g^−1^ soft tissue) in this study were measured in tissues but no toxicities were predicted by the model (e.g., ESP‐B and C; Fig. 4; Supporting Information Fig. S1). These discrepancies coincide with low or undetectable *A. catenella* concentrations measured by the ESP (Fig. 2) and may be attributed to bound toxins, local, patchy, or low‐intensity blooms, or spatial separation between the ESP deployment locations and toxicity measurements. The first hypothesis is unlikely because *M. edulis* is known to rapidly depurate PSTs, particularly at low to moderate toxicity levels (e.g., the depuration rate in *S. giganteus* is 20‐fold slower; *see* Table 4 in Bricelj and Shumway 1998).

While the one‐compartment model effectively fits the observed data, variation of fits was noted among the models. Notably, γ was not a primary driver for this variation, as similar fits were found with variable or fixed depuration rates (e.g., M1 vs. M3). This aligns with studies that found that PST accumulation models for *M. edulis* (Silvert and Cembella 1995) and *M. galloprovincialis* (Blanco et al. 2003) were not highly sensitive to γ. In addition, optimal γ values fell within a narrow range and were consistent with values reported in in vitro and field studies (Supporting Information Table S1; Bricelj and Shumway 1998).

The toxin uptake parameter β had a greater influence on the model fits than the depuration parameter and averaged ca. 0.20–0.30 *μ*g STX eq. 100 g^−1^ tissue L cell^−1^ d^−1^, with similar ranges for both local and global fits. Directly comparable uptake rates from similar models in equivalent units were not reported in the literature; thus, we calculated them when data permitted. We used field data on cell concentration and *M. edulis* toxicity in Silvert and Cembella (1995) and averaged values of weight‐standardized clearance rate (i.e., calculated for a standard‐sized mussel of 1 g wet weight of soft tissues) from Bricelj et al. (1990) to calculate an uptake parameter of 1.1 × 10^−2^ ± 9.5 × 10^−3^ *μ*g STX eq. 100 g^−1^ tissue L cell^−1^ d^−1^ (average ± SD). Using data from Tang et al. (2021) from a study of *M. scoruscus* we calculated uptake of 1.4 × 10^−5^ ± 1.0 × 10^−5^ *μ*g STX eq. 100 g^−1^ tissue L cell^−1^ d^−1^. These calculated values span three orders of magnitude and all were lower than those obtained in the present study.

Our analysis indicated that a range of combinations of β and γ parameters could yield similarly good model fits (Figs. 5 and 6). This result illustrates uncertainty in the model optimization based on the ESP and toxicity data alone and suggests that biological constraints on the parameters could help limit the uncertainty. The uptake parameter serves as a mathematical representation of several feeding processes in mussels, including clearance rate, ingestion rate [i.e., (clearance rate × particle concentration − pseudofeces production)], and absorption rate (ingestion rate × absorption efficiency of algal cells in the gut) (Bayne et al. 1993; Bayne 2017). Variability in these feeding processes could be attributed to a range of biological and environmental factors.

*Mytilus edulis* nerves are relatively insensitive to PSTs toxicity compared to other North American suspension‐feeding bivalves (Bricelj and Shumway 1998). This allows mussels to continue feeding during *A. catenella* toxic blooms, as shown in laboratory studies (Bricelj and Shumway 1998), at cell toxicity higher than that of isolates from the GoM (Anderson et al. 1994). Field studies showed that *A. catenella* strains from coastal regions of the GoM exhibit consistent toxin profiles and quantities across locations and years (Anderson et al. 1994). Therefore, cell toxicities are not expected to cause marked reduction in feeding rates of *M. edulis* populations in the GoM, and variability in cell ingestion rates cannot be readily attributed to the variation in mussel CR in response to *Alexandrium* specific toxicity.

Many other factors can modulate bivalve feeding rates in the natural environment where they are exposed to mixed suspensions. Seston characteristics and phytoplankton species composition are known to influence mussel clearance rate and thus algal ingestion rate. Seston concentrations in coastal habitats of *M. edulis* in the GoM range from 0.1 to 25 mg dry weight (DW) L^−1^ in the Kennebec River estuary, Casco Bay (Kistner and Pettigrew 2001). However, episodic events such as high river discharge, spring tides, and storms could elicit local transient increases in seston concentrations. The seston concentration greater than ~ 5 mg DW L^−1^ induces pseudofeces production in *M. edulis* (Widdows et al. 1979). While both changes in clearance rate and pseudofeces production provide mussels with mechanisms to regulate the ingestion of organic matter, they can also yield highly variable particulate organic matter ingestion and consequently variable toxin uptake rates. Additionally, mussel collections by monitoring programs are typically from intertidal beds, and the fraction of time animals spend submerged vs. out of water varies among locations. This can affect both uptake and depuration of PSTs and may contribute to variability in fits among GoM stations.

Temperature is an important environmental factor affecting the clearance rate of poikilotherms, including bivalve suspension feeders. However, numerous laboratory studies have shown that clearance rate and thus ingestion rate of *M. edulis* remains relatively constant over a wide range of acclimation temperatures, for example, 10°C to 15°C (Widdows and Bayne 1971). Similarly, Moroño et al. (2001) found no significant effect of temperature on the PST uptake rate of *M. galloprovincialis* between 13.6°C and 18.4°C. Attempts to incorporate abiotic (e.g., temperature, salinity) and biotic (e.g., cell concentration) parameters into toxin compartment‐model equations have yielded mixed results (Blanco et al. 1997; Moroño and Blanco 1997; Tang et al. 2021).

The accumulation rate of PSTs by *M. edulis* is strongly influenced by mussel size/weight (Bricelj and Shumway 1998; Moroño et al. 2001). Body size effects are only partially corrected by calculation of weight‐specific bivalve toxicities used in the context of monitoring programs, given that weight‐specific ingestion rate (and thus toxicity per unit body weight) decreases with increasing body size (Bayne and Newell 1983). Maine DMR records the total wet tissue weight and the corresponding number of mussels contributing to this weight for subsequent toxin extraction (Bryant J. Lewis, ME DMR, pers. comm.), but not the individual shell dimension of individual mussels sampled.

### Model implications for toxicity monitoring

While the approach of this study focuses on modeling toxin accumulation, similar ESP‐based technology has been used for other HAB species. For example, Doucette et al. (2009) used ESPs to detect *Pseudo‐nitzschia* cells and their domoic acid cell toxicity in Monterey Bay, California, using the enzyme‐linked immunosorbent assay (ELISA). This approach was later used to describe *Pseudo‐nitzschia* bloom dynamics in the eastern Pacific (Ryan et al. 2017; Moore et al. 2021). Although not incorporated into predictive models, these applications highlight the capacity of autonomous systems to support HAB monitoring for multiple toxic species and toxin groups, particularly in remote regions where coastwide closures are implemented.

All the models in the present study were initialized using Maine DMR toxicity values for each site and subsequently provided daily toxicity updates for several weeks without producing false negatives and only a few false positives, making them potentially useful for public health authorities and end‐users (e.g., aquaculture and tourism industries). This approach aligns with recent stakeholder‐driven initiatives that emphasize the need for site‐specific, near‐term, and toxin‐focused forecasts to support shellfish farmers and coastal managers (Record et al. 2022). These models have the potential to complement traditional PSP monitoring programs by providing direct links from toxic cell concentration to shellfish toxicity at important spatial and temporal scales, thus addressing limitations of traditional sampling strategies, particularly in less populated, remote areas, where monitoring costs are higher and sampling at adequate timescales is logistically impractical. The preferred model (*R* ^2^ of 0.85 and lowest AIC value) for site‐specific monitoring uses locally fitted uptake parameters with a fixed depuration parameter (M3). While this approach requires recalculating uptake parameters for each ESP deployment, monitoring site, and year, it can be useful where a strong relationship exists between offshore mooring and mussel toxicity at coastal stations. ESPs deployed in coastal embayments could provide continuous, site‐specific toxicity data, making them valuable tools for early‐warning systems. This model is especially relevant in areas with shellfish farming or recreational harvesting, where localized monitoring is limited (M1). Models using globally fitted parameters (M2 and M4) may be more broadly applicable and require less site‐specific calibration.

### Limitations

Until further studies are undertaken on the applicability of ESP‐based models to complement shellfish monitoring strategies, several limitations can already be highlighted. One is cost, as the ESP instrument is expensive, as is the surface buoy and hose system used for these deployments (around 300 K$). The instrument/mooring assembly is large and requires a sizeable vessel for deployment and recovery, further increasing costs. An alternative approach could be the deployment of ESPs on docks and piers to reduce costs, coupled with Models 1 and 3 for site‐specific monitoring.

Stock et al. (2007) used a coupled physical–biological model to simulate *A. catenella* blooms in the GoM showing that unresolved small spatiotemporal‐scale variability, such as patchiness in nutrient concentrations and bloom dynamic processes (e.g., grazing, encystment), contributed to the poor fits of the model. Overall, such variation could cause differences in the relationship between ESP data and shellfish toxicity among locations.

### Perspective on the one‐compartment model for toxin monitoring

A promising perspective would be to couple the one‐compartment toxicity model with a physical–biological model that forecasts *Alexandrium* abundance to predict toxicity in coastal waters. A physical–biological model combining regional circulation with *A. catenella* cyst distributions and cell growth has been developed to simulate bloom dynamics at weekly to seasonal scales in the GoM (McGillicuddy et al. 2011; https://northeasthab.whoi.edu/hab-hub/). This model calculates the spatial and temporal distributions of *A. catenella* cell concentrations but does not directly calculate shellfish toxicity that is of greatest interest to resource managers. Pairing the physical–biological model cell concentrations with a one‐compartment toxicity model could aid in forecasting impacts of *A. catenella* blooms. To further improve model accuracy, mussel toxicity could potentially be assimilated into the physical–biological model to constrain *A. catenella* cell concentrations (Anderson et al. 2013).

## Author Contributions

Sylvain Gaillard: Formal analysis; Writing—Original Draft; Writing—Review and Editing; Visualization. David K. Ralston: Conceptualization; Methodology; Software; Formal analysis—Original Draft; Writing—Review and Editing; Visualization. V. Monica Bricelj: Formal analysis; Writing—Original Draft; Writing—Review and Editing. Roman Marin: Conceptualization; Methodology. Bruce Keafer: Conceptualization; Methodology; Investigation. Dennis J. McGillicuddy Jr.: Conceptualization; Methodology; Investigation; Writing—Original Draft; Funding acquisition. Donald M. Anderson: Conceptualization; Methodology; Formal analysis; Investigation; Writing—Original Draft; Writing—Review and Editing; Supervision; Funding acquisition.

## Conflicts of Interest

None declared.

## Supporting information


**Table S1.** Field or laboratory studies modeling paralytic shellfish toxin dynamics in commercially important mussel species (*Mytilus* spp.). SD: standard deviation; n.d.: not determined; n.a.: not available. Summary values of uptake and depuration parameters are expressed as the averaged mean variable values for each study (when data were available).
**Table S2.** Environmental Sample Processor (ESP) deployment locations.
**Table S3.** Maine Department of Marine Resources (DMR) blue mussel (*Mytilus edulis*) sampling stations used in this study.
**Table S4.** Model *R* ^2^ (Pearson's correlation coefficient squared) and free parameter values of the local fits of the 15 selected good fits (Figs. 3 and 4; Supporting Information Tables S2 and S3). ESP: Environmental Sample Processor (locations, represented by letters; stations are indicated in Fig. 1); M0: model 0; M0L: model 0 lagged; M1: model 1; M2: model 2; M3: model 3; M4: model 4; SD: standard deviation; ns: non‐significant difference; * *p* ≤ 0.05; ** 0.001 < *p* ≤ 0.01; *** *p* ≤ 0.001. β and γ: toxin uptake and depuration parameters; *R* ^2^: Pearson's correlation coefficient squared.
**Fig. S1.** Correlation plots of the observed mussel toxicity [μg saxitoxin (STX) equivalent 100 g^−1^ soft tissue] and observed *Alexandrium catenella* (Alex) concentrations (cells L^−1^) for the models M0 and M0L (Table 1 and Supporting Information Table S4). *R* ^2^: Person's correlation coefficient squared.
**Fig. S2.** Time series of observed paralytic shellfish toxin in blue mussel (*Mytilus edulis*) tissues [μg saxitoxin (STX) equivalents (eq.) 100 g^−1^ soft tissue] sampled at coastal Maine Department of Marine Resources (DMR) stations (Fig. 1), and modeled toxicity (μg STX eq. 100 g^−1^ soft tissue) (Table 1 and Supporting Information Table S4). Dashed line represents the regulatory limit (i.e., 80 μg STX eq. 100 g^−1^ soft tissue). β: toxin uptake parameter; γ: toxin depuration parameters; ESP: Environmental Sample Processor; M1, M2, M3, and M4: models 1 through 4.

## Data Availability

The data that support the findings of this study are openly available in Zenodo at https://doi.org/10.5281/zenodo.17737458.
