# Supplementary material for: Model predicts coastal paralytic shellfish toxicity in mussels from in situ phytoplankton concentrations
Source: Limnol Oceanogr. 2026 Sep 14;71(9):e70502. doi: 10.1002/lno.70502 (PMC13596099; doi:10.1002/lno.70502)
Supplement: Supplementary file 1 [file LNO-71-e70502-s001.docx]

**Supporting Information for:**

**Title:** Model predicts coastal mussel toxicity from *in situ* phytoplankton concentrations

**Authors:** Sylvain Gaillard^1*^, David K. Ralston^1^, V. Monica Bricelj^2^, Roman Marin III^3^, Bruce Keafer^1^, Dennis J. McGillicuddy Jr.^1^, Donald M. Anderson^1^.

^*^ Corresponding author [sylvain.gllrd@gmail.com](mailto:sylvain.gllrd@gmail.com)

^1^ Woods Hole Oceanographic Institution, Woods Hole MA 02543 USA

^2^ TechMar Research Inc., Brooklyn, NY, USA

^3^ Monterey Bay Aquarium Research Institute, Moss Landing, CA, USA

**Table S1**. Field or laboratory studies modeling paralytic shellfish toxin dynamics in commercially important mussel species (*Mytilus* spp.). SD: standard deviation; n.d.: not determined; n.a.: not available. Summary values of uptake and depuration parameters are expressed as the averaged mean variable values for each study (when data were available).

| Bivalve mollusk species | Dinoflagellate species, exposure source and concentrations (cells L^-1^) | Type of model | Uptake parameter | Depuration parameter | Comments | Reference |
| --- | --- | --- | --- | --- | --- | --- |
| *Mytilus edulis* (blue mussel) | *Alexandrium catenella*, bloom, 10^5^ to 10^6^ | One-compartment or two-compartment | 1.1 × 10^-2^ ± 9.5 × 10^-3^ (average ± SD) 9.0 × 10^-4^ (min) to 2.6 × 10^-2^ (max) μg STX eq. 100 g^-1^ tissue L cell^-1^ d^-1^ ^a^ | Constant: 0.24 and 0.40 d^-1^ | One-compartment model provided a "useful representation of toxin dynamics", but the fit was "better" using a two-compartment model. The modeled accumulation was not "very sensitive" to modification of the depuration parameter. | Silvert and Cembella 1995 |
| *M. edulis* | *A. catenella*, bloom, 1.0 × 10^2^ (average) and 6.8 × 10^3^ (max). | One-compartment | Variable:  3.1 × 10^-2^ ± 1.7 × 10^-2^ (average ± SD) 9.0 × 10^-3^ (min) to 7.2 × 10^-2^ μg STX eq. 100 g^-1^ tissue L cell^-1^ d^-1^ | Constant: 0.15 or variable: 0.16 ± 9.3 × 10^-2^ (average ± SD) 3.0 × 10^-2^ (min) to 0.35 (max) | Moderate to high *R^2^* depending on parameter values. Fit was sensitive to the uptake but not the depuration parameters and was higher when using locally fitted parameters. | This study |
| *M. galloprovincialis* (Mediterranean mussel) | *Gymnodinium catenatum*, bloom, n.d. | One or two-compartment | n.d. | Constant: 0.15 or 0.21 d^-1^ | Higher *R*^2^ using two-compartment model (0.89) than one-compartment model (0.45). *R*^2^ was sensitive to incorporation of cellular toxin concentration parameter and to low *A. catenella* concentrations. | Moroño and Blanco 1997 |
| *M. galloprovincialis* | *G. catenatum*, bloom, n.d. | Two-compartment - depuration model | n.a. | Constant or variable: -8.0 × 10^-2^ (min) to 0.27 (max). Best fit: 0.15 d^-1^ | Two-compartment model provided a “better” fit than one-compartment model. “Better” fit when depuration was adjusted with environmental (biotic and abiotic) variables. High *R*^2^ of 0.92 | Blanco et al. 1997 |
| *M. galloprovincialis* | *A. minutum*, culture, 1 × 10^6^ | One or two-compartment | Constant: 4.6 L.h^-1^ | Constant: 0.15 or variable: 0 (min) to 0.28 (max) d^-1^ | One-compartment with constant uptake and depuration, and transformation of toxins, provided the “best” fit. Variation in depuration rate did not significantly increase the fit. | Blanco et al. 2003 |
| *M. galloprovincialis* | *G. catenatum*, bloom and culture, >10^3^ | One-compartment - depuration model | n.a. | Fitted according to the toxin: 4.8 x10^-2^ (min) to 8.0 × 10^-2^ (max) (*in vitro* exp) and 7.1 × 10^-2^ (min) to 0.15 (max) d^-1^ (*in vivo* exp). Average: 8.0 × 10^-2^ d^-1^ | Low *R*^2^. May be due to the “intraspecific variation” of toxin accumulation. | Botelho et al. 2010 |
| *M. galloprovincialis* | *G. catenatum*, culture, 9.1 × 10^4^ cells^-1^ d^-1^ individual^-1^ | One-compartment | n.d. | Fitted according to the toxin, temperature, and pH: 6.0 × 10^-2^ to 0.90 d^-1^. Average: 0.31 d^-1^ | High *R*^2^ averaging 0.84. Lowest depuration rate in warm-acclimated (24 °C) animals vs. control (19 °C) (0.14 ± 0.11 vs. 0.26 ± 0.20, average ± SD). | Braga et al. 2018 |
| *M. coruscus* (Korean mussel) | *A. catenella*, culture, 5.6 × 10^3^ to 1.0 × 10^7^ | One-compartment | 1.4 × 10^-5^ ± 1.0 × 10^-5^ (average ± SD) 4.0 × 10^-6^ (min) to 3.6 × 10^-5^ (max) μg STX eq. 100 g^-1^ tissue L cell^-1^ d^-1^ ^a^ | 0.25 ± 0.15 (average ± SD) 6.0 × 10^-2^ (min) to 0.53 (max) | High *R*^2^ averaging 0.89. Depuration rates varied with temperature (0.18 ± 0.058 at control (24 °C) vs. 0.32 ± 0.18 at high temperature (30 °C)) and phytoplankton concentration (0.28 ± 0.14 at low vs. 0.22 ± 0.17 at high concentration). | Tang et al. 2021 |
| Summary (average ± SD) | | | 0.014 ± 0.016 μg STX eq. 100 g^-1^ tissue L cell^-1^ d^-1^ | 0.16 ± 0.10 d^-1^ |  |  |

^a^ calculated from the authors data

**Table S2**. Environmental Sample Processor (ESP) deployment locations

| Year | ESP | Latitude (dms) | Longitude (dms) |
| --- | --- | --- | --- |
| 2014 | ESP-A | 43°10'36.486"N | 70°26'35.610"W |
|  | ESP-B | 43°41'13.836"N | 69°58'36.492"W |
|  | ESP-C | 43°48'10.536"N | 69°27'26.946"W |
| 2017 | ESP-D | 44°34'4.860"N | 67°17'40.560"W |
| 2019 | ESP-E | 44°34'4.800"N | 67°17' 420"W |
|  | ESP-F | 44°42'11.736"N | 66°29'8.244"W |

**Table S3**. Maine Department of Marine Resources (DMR) blue mussel (*Mytilus edulis*) sampling stations used in this study.

| Year | Toxin station | Latitude (dms) | Longitude (dms) |
| --- | --- | --- | --- |
| 2014 | York River | 43°7'38.172"N | 70°38'39.336"W |
|  | Basin Point | 43°44'18.528"N | 70°2'36.348"W |
|  | Potts Point | 43°43'50.304"N | 70°1'32.0160"W |
|  | Head Beach | 43°43'01.6"N | 69°50'60.0"W |
|  | Christmas Cove | 43°50'51.216"N | 69°33'25.272"W |
|  | Port Clyde | 43°55'30.936"N | 69°15'32.400"W |
| 2017 | Gove Point | 44°54'19.620"N | 67°3'22.356"W |
|  | Gleason Cove | 44°58'15.0240"N | 67°3'9.144"W |
|  | Lubec Channel | 44°51'32.0760"N | 66°58'49.00800"W |
| 2019 | Gove Point | 44°54'19.620"N | 67°3'22.356"W |
|  | Gleason Cove | 44°58'15.0240"N | 67°3'9.144"W |
|  | Lubec Channel | 44°51'32.0760"N | 66°58'49.00800"W |

**Table S4**. Model *R*^2^ (Pearson’s correlation coefficient squared) and free parameter values of the local fits of the 15 selected good fits (Figs. 3, 4, Supporting Information Tables S2-3). ESP: Environmental Sample Processor (locations, represented by letters; stations are indicated in Fig. 1); M0: model 0; M0L: model 0 lagged; M1: model 1; M2: model 2; M3: model 3; M4: model 4; SD: standard deviation; ns: non-significant difference; * *p* ≤ 0.05; ** 0.001 < *p* ≤ 0.01; *** *p* ≤ 0.001. $\beta$ and $\gamma$: toxin uptake and depuration parameters; *R^2^*: Pearson’s correlation coefficient squared.

| Year | ESP | Mussel toxicity station | Distance between pairs (km) | M0 *R*^2^ | M0L *R*^2^ | M0L n^o^ days | M1 $\beta$ | M1 $\gamma$ | M1 *R*^2^ | M2 *R*^2^ | M3 $\beta$ | M3 *R*^2^ | M4 *R*^2^ |
| --- | --- | --- | --- | --- | --- | --- | --- | --- | --- | --- | --- | --- | --- |
| 2014 | A | York River | 17 | 0.70^*^ | 0.89^**^ | 1 | 0.015 | 0.17 | 0.86^**^ | 0.83^**^ | 0.015 | 0.86^**^ | 0.86^**^ |
| 2014 | B | Basin Point | 7.8 | 0.35^ns^ | 0.52^ns^ | 2 | 0.054 | 0.33 | 0.67^*^ | 0.38^ns^ | 0.024 | 0.51^ns^ | 0.51^ns^ |
| 2014 | B | Potts Point | 6.2 | 0.37^ns^ | 0.57^ns^ | 2 | 0.054 | 0.35 | 0.75^*^ | 0.37^ns^ | 0.024 | 0.53^ns^ | 0.53^ns^ |
| 2014 | C | Head Beach | 33 | 0.96^**^ | 0.75^ns^ | 1 | 0.066 | 0.13 | 0.96^**^ | 0.95^**^ | 0.072 | 0.96^**^ | 0.96^**^ |
| 2014 | C | Christmas Cove | 9.4 | 0.60^ns^ | 0.65^ns^ | 6 | 0.033 | 0.19 | 0.82^*^ | 0.68^ns^ | 0.027 | 0.79^*^ | 0.78^*^ |
| 2014 | C | Port Clyde | 21 | 0.59^ns^ | 0.82^*^ | 7 | 0.012 | 0.03 | 0.90^*^ | 0.83^*^ | 0.024 | 0.74^ns^ | 0.74^ns^ |
| 2017 | D | Gove Point | 42 | 0.04^ns^ | 0.72^**^ | 7 | 0.009 | 0.09 | 0.91^***^ | 0.90^***^ | 0.012 | 0.90^***^ | 0.90^***^ |
| 2017 | D | Gleason Cove | 49 | 0.02^ns^ | 0.81^**^ | 7 | 0.03 | 0.11 | 0.99^***^ | 0.93^***^ | 0.039 | 0.98^***^ | 0.93^***^ |
| 2017 | D | Lubec Channel | 41 | 0.06^ns^ | 0.87^***^ | 2 | 0.021 | 0.15 | 0.94^***^ | 0.88^***^ | 0.021 | 0.94^***^ | 0.94^***^ |
| 2019 | E | Gove Point | 46 | 0.01^ns^ | 0.69^*^ | 7 | 0.021 | 0.09 | 0.77^**^ | 0.78^**^ | 0.03 | 0.58^*^ | 0.57^*^ |
| 2019 | E | Gleason Cove | 53 | 0.28^ns^ | 0.42^ns^ | 3 | 0.012 | 0.05 | 0.41^ns^ | 0.42^ns^ | 0.048 | 0.33^ns^ | 0.16^ns^ |
| 2019 | E | Lubec Channel | 46 | 0.97^**^ | 0.98^**^ | 2 | 0.027 | 0.15 | 0.98^**^ | 0.96^**^ | 0.027 | 0.98^**^ | 0.98^**^ |
| 2019 | F | Gove Point | 50 | 0.01^ns^ | 0.78^**^ | 1 | 0.021 | 0.13 | 0.97^***^ | 0.97^***^ | 0.021 | 0.95^***^ | 0.95^***^ |
| 2019 | F | Gleason Cove | 54 | 0.92^*^ | 0.94^*^ | 1 | 0.066 | 0.29 | 0.98^*^ | 0.85^ns^ | 0.027 | 0.91^*^ | 0.89^ns^ |
| 2019 | F | Lubec Channel | 43 | 0.93^**^ | 0.91^*^ | 4 | 0.045 | 0.17 | 0.76^ns^ | 0.82^*^ | 0.039 | 0.78^*^ | 0.75^ns^ |
|  |  | Overall/mean (± SD) | 35  (± 17) | 0.12^***^ | 0.45^***^ | 2 | 0.032  (± 0.020) | 0.162 (± 0.095) | 0.90^***^ | 0.71^***^ | 0.030  (± 0.015) | 0.85^***^ | 0.69^***^ |

*R^2^ = 0.12*

*R^2^ = 0.45*

Fig. S1. Correlation plots of the observed mussel toxicity [µg saxitoxin (STX) equivalent 100g^-1^ soft tissue] and observed *Alexandrium catenella* (Alex) concentrations (cells L^-1^) for the models M0 and M0L (Table 1 and Supporting Information Table S4). *R^2^*: Person’s correlation coeeficient squared.

Fig. S2. Time series of observed paralytic shellfish toxin in blue mussel (*Mytilus edulis*) tissues [µg saxitoxin (STX) equivalents (eq.) 100g^-1^ soft tissue] sampled at coastal Maine Department of Marine Resources (DMR) stations (Fig. 1), and modeled toxicity (µg STX eq. 100g^-1^ soft tissue) (Table 1 and Supporting Information Table S4). Dashed line represents the regulatory limit (i.e., 80 µg STX eq. 100g^-1^ soft tissue). $\beta$: toxin uptake parameter; $\gamma$: toxin depuration parameters; ESP: Environmental Sample Processor; M1, M2, M3, and M4: models 1 through 4.
